# Viscoelastic properties of biopolymer hydrogels determined by Brillouin spectroscopy: A probe of tissue micromechanics

**DOI:** 10.1126/sciadv.abc1937

**Published:** 2020-10-30

**Authors:** Michelle Bailey, Martina Alunni-Cardinali, Noemi Correa, Silvia Caponi, Timothy Holsgrove, Hugh Barr, Nick Stone, C. Peter Winlove, Daniele Fioretto, Francesca Palombo

**Affiliations:** 1 University of Exeter, School of Physics and Astronomy, Exeter EX4 4QL, UK.; 2 University of Perugia, Department of Physics and Geology, Perugia I-06123, Italy.; 3 CNR-IOM–Istituto Officina dei Materiali–Research Unit in Perugia, Department of Physics and Geology, University of Perugia, Perugia I-06123, Italy.; 4 University of Exeter, School of Engineering, Exeter EX4 4QF, UK.; 5 Gloucestershire Royal Hospital, Gloucester GL1 3NN, UK.

## Abstract

Many problems in mechanobiology urgently require characterization of the micromechanical properties of cells and tissues. Brillouin light scattering has been proposed as an emerging optical elastography technique to meet this need. However, the information contained in the Brillouin spectrum is still a matter of debate because of fundamental problems in understanding the role of water in biomechanics and in relating the Brillouin data to low-frequency macroscopic mechanical parameters. Here, we investigate this question using gelatin as a model system in which the macroscopic physical properties can be manipulated to mimic all the relevant biological states of matter, ranging from the liquid to the gel and the glassy phase. We demonstrate that Brillouin spectroscopy is able to reveal both the elastic and viscous properties of biopolymers that are central to the structure and function of biological tissues.

## Teaser

Gelatin hydrogels cover a wide range of microenvironments that are relevant for bioapplications of Brillouin elastography.

## INTRODUCTION

The mechanical properties of living cells and tissues are essential to their physiological function and, on a microscopic scale, they determine many aspects of cellular activity (*1*–*3*). These properties are largely determined by the cytoskeleton in the cell and by networks of collagen and elastin fibers in the extracellular matrix. Classical mechanical testing has provided a basis of understanding how the composition and organization of the networks in specific tissues yield the requisite mechanical properties and has demonstrated functionally relevant changes in diseases ranging from atherosclerosis to osteoarthritis. However, research interest in these diseases has now moved to the subcellular level, and this has generated an urgent need to characterize the mechanical properties of tissues on these length scales. In this framework, Brillouin microspectroscopy has emerged as a compelling tool in biomedical sciences. The technique is based on Brillouin light scattering (BLS), which is an acoustic process arising from the interaction of light with thermally driven acoustic phonons at high frequencies (*4*, *5*). It probes microelasticity and viscosity through the measurement of the longitudinal elastic modulus and acoustic wave attenuation, providing a unique insight into mechanical properties on a microscale.

Early work determined that this technique could provide a new contrast mechanism for the study of live cells (*6*–*10*) and organisms (*11*–*13*), histological tissue sections (*14*, *15*), and cornea (*16*, *17*), as well as providing new insights into mechanobiology (*18*, *19*). BLS has also been used to measure the complete elasticity tensor and mechanical anisotropy of fibrous proteins (*20*, *21*), the only technique capable of measuring this. For collagen and elastin fibers (*21*–*25*), the longitudinal modulus was found to be many orders of magnitude higher than the moduli determined by more classical engineering approaches. This discrepancy is presumed to reflect the different spatiotemporal scales of the two types of measurement [although nanomechanical testing and molecular dynamics (MD) simulations of collagen-like triple helix yield a Young’s modulus in the gigapascal range (*26*)], as well as that between the longitudinal modulus and the shear and Young’s moduli that are more widely used in bioengineering. This distinction has been considered in recent literature (*27*–*29*). However, a complicating factor is the contribution of water both to tissue/cell biomechanics and to the Brillouin spectrum. The former has previously been established through the use of pore-elastic models (*30*, *31*); however, recent work suggests that the contribution of water is far more complex, with cell mechanics heavily affected by water (*32*–*34*), in particular osmotic-induced volume change affects cell stiffness (*32*) and deformability (*34*), fluid flow through cell-cell gap junctions induces mechanical pattern formation (*33*), and cell migration in confinement is driven by cell volume regulation (*35*). The contribution of water to the Brillouin spectrum is still a subject of controversy with some reports that in highly hydrated fluids, simulating some aspects of the cell cytoplasm, the frequency shift of the Brillouin peak is determined by modes generated in the water phase (*36*, *37*).

In light of this, we sought to explore the information content of the Brillouin spectrum of gelatin (denatured type I collagen) gels, which are simple model systems derived from the most ubiquitous structural protein, and compare those with real tissue samples. By varying the polymer concentration, it is possible to cover a wide range of static and dynamic macroscopic mechanical moduli that replicate those of many biological tissues. We show that for low polymer concentrations, the Brillouin linewidth, which is a viscosity indicator, is much more sensitive to concentration than the frequency shift, thus making a full band shape analysis necessary to assess viscoelasticity in biological samples. This microscopic viscosity is key to transport processes in cells and regulates the rate of biological processes at the nanoscale, for example, in cellular organelles (*38*). We further show that the local increase in viscosity of the gelatin-water liquid phase is mainly due to a factor 1.9 retardation of the dynamics of water at the interface with gelatin, comparable to the effect of hydrophobic hydration for a large class of biomimetic molecule mixtures. By decreasing the water content, we observe a liquid-glass transition that drives the system toward the solid-like behavior, typical of many tissues such as tendon and bone. We show that articular cartilage presents high- and low-frequency modes from collagen fiber bundles and matrix-dispersed collagen, respectively, at those frequencies observed for the model gelatins at high and low polymer concentration. A glass transition analogous to that observed for colloidal systems has previously been observed in the crowded cytoplasm space of compressed cells and is found to be universal across cell types (*39*). In addition, proteins that normally form liquid-like phases, in the case of diseases such as Alzheimer’s, Parkinson’s, and amyotrophic lateral sclerosis (ALS), end up taking more solid-like properties (*40*). Other biogels, namely actin gels, present soft matter and glassy physics, in particular ageing-dependent viscoelastic properties (*41*). Therefore, results presented here cover a broad range of microenvironments that are relevant for biological applications of Brillouin elastography in living systems.

These results demonstrate that the Brillouin-derived viscoelastic parameters of gelatin hydrogels as model systems for protein networks are dominated by the interaction of solute with the solvent relaxation dynamics. Moreover, they suggest an analogy to a percolating colloidal suspension approaching the glass transition controlled by concentration (*39*), revealed here by Brillouin spectroscopy. This work provides a framework to characterize the viscoelastic properties of protein networks across a broad range of physical conditions and corroborates Brillouin spectroscopy as a reliable tool to characterize the biomechanical changes in complex systems such as biological tissues. It discloses new important applications for Brillouin spectroscopy in fundamental research, tissue engineering, and clinical diagnosis.

## RESULTS AND DISCUSSION

### High hydration

**Fig. 1:**
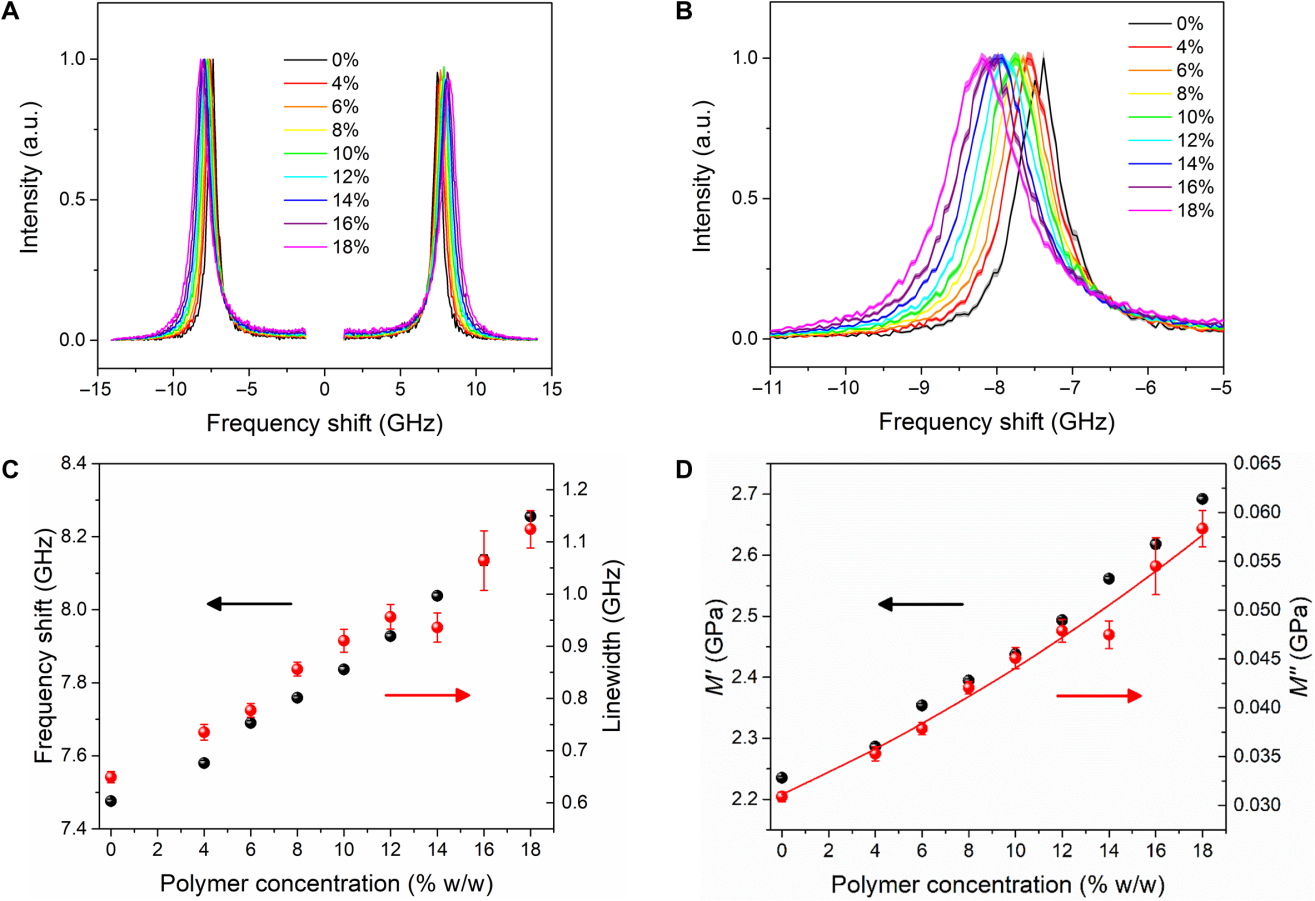
High hydration Brillouin spectroscopy. Dependencies of (**A**) Brillouin spectra, (**B**) Stokes peak, (**C**) frequency shift and linewidth, and (**D**) storage and loss moduli of gelatin gels on polymer concentration. Spectra are normalized to the maximum of the Stokes peak. Full symbols, experimental data; error bars, standard error, i.e., square root of number of counts; red line, linearized model (see text); and a.u., arbitrary unit.

Brillouin spectra of collagen gels contain only a single sharp peak (with Stokes and anti-Stokes components; see Fig. 1A), indicating that the gels are homogeneous on the phonon wavelength scale (ca. 0.3 μm for 532-nm excitation). With increasing polymer concentration, there is an increase in both the Brillouin frequency shift ω_B_ and linewidth Γ_B_ (Fig. 1B), in line with previous observations (*42*, *43*). The values of ω_B_ and Γ_B_ were derived from fitting the peaks to a damped harmonic oscillator (DHO) function (see fig. S1; Fig. 1C). An observed Brillouin shift of the order of 8 to 9 GHz reproduces well the situation of the cornea in highly hydrated conditions (*16*). The Lorentz-Lorenz equation (which predicts the ratio of density-to-refractive index squared to be approximately constant) was found to be true for the samples tested (see Materials and Methods and fig. S2), so the changes in ω_B_ and Γ_B_ are unambiguously assigned to an increase in the storage and loss moduli (Fig. 1D).

**Fig. 2:**
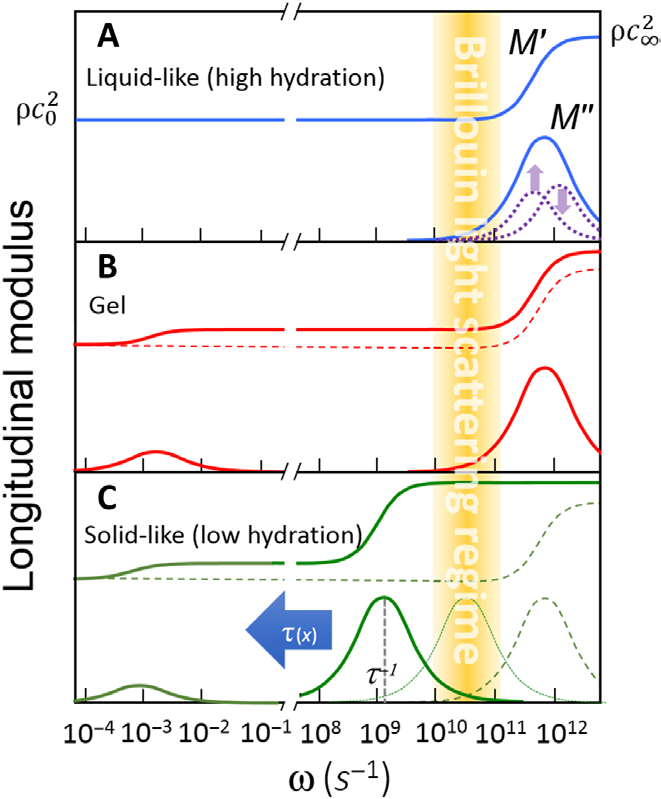
Dispersion in longitudinal storage and loss moduli. Schematic diagram of the dispersion in longitudinal storage modulus *M*^′^(ω) (top curves) and loss modulus *M*^′′^(ω) (bottom curves) in the (**A**) high hydration limit, (**B**) gel phase, and (**C**) low hydration limit. Yellow shaded area denotes the Brillouin region [see also (*5*)].

Changing polymer concentration has the largest effect on the Brillouin linewidth (89% variation; Fig. 1, C and D), which reflects variations in the microscopic viscosity of the gel. This variation is attributed to the restricted mobility of water, particularly in the first hydration shell of the polymer molecules (see Materials and Methods). This mechanism is shown in the schematic diagram of the frequency dependence of the storage modulus *M*′(ω) and loss modulus *M*′′(ω) at different polymer concentrations (Fig. 2). In the dilute limit (Fig. 2A), the modulus at Brillouin frequencies (shadowed area) is that of a simple liquid, with a “relaxed” storage modulus ${M_{0}}^{\prime }=\rho c_{0}^{2}$, where $c_{0}=\frac{\omega_{B}}{q}$ is the adiabatic sound velocity, and a loss modulus *M*^′′^ = ω_B_*b*, where $b=\rho \frac{\Gamma_{B}}{q^{2}}$ is the longitudinal kinematic viscosity (ρ, sample’s mass density; *q*, exchanged wave vector, *q* = 4π*n*/λ in backscattering geometry; *n*, sample’s refractive index; and λ, excitation wavelength, 532 nm).

While the system is in a liquid-like state, the structural relaxation responsible for the increase in modulus up to the solid-like value ${M_{\infty }}^{\prime }=\rho c_{\infty }^{2}$ occurs on a picosecond time scale, i.e., at much higher frequency than the Brillouin peak, giving rise to a linear relationship between loss modulus and frequency, *M*′′(ω) = ρω*b*, where the gradient gives the viscosity *b*. In the case of highly diluted polymer, water may be considered as having two phases, “free” (or bulk) water and “hydration” water (interacting more strongly with the polymer chains). In this state, the observed increase in Γ_B_ or *M*^′′^ with increasing polymer concentration (Fig. 1, C and D) can be attributed to an increase in *b* due to two contributions to the relaxation process (dotted lines in Fig. 2A), one from bulk and the other (retarded) from hydration water, which increases linearly with polymer concentration. Using this model and a hydration number derived from simple geometric arguments (10,071; see Materials and Methods), we obtain an average retardation of the order of 1.9 (fig. S3) and values of *M*^′′^ that match the experimental data (red line in Fig. 1D). This value of retardation is in line with results of previous measurements and theoretical analyses of hydrophobic hydration that are appropriate to the structure of the collagen molecule (*44*).

**Fig. 3:**
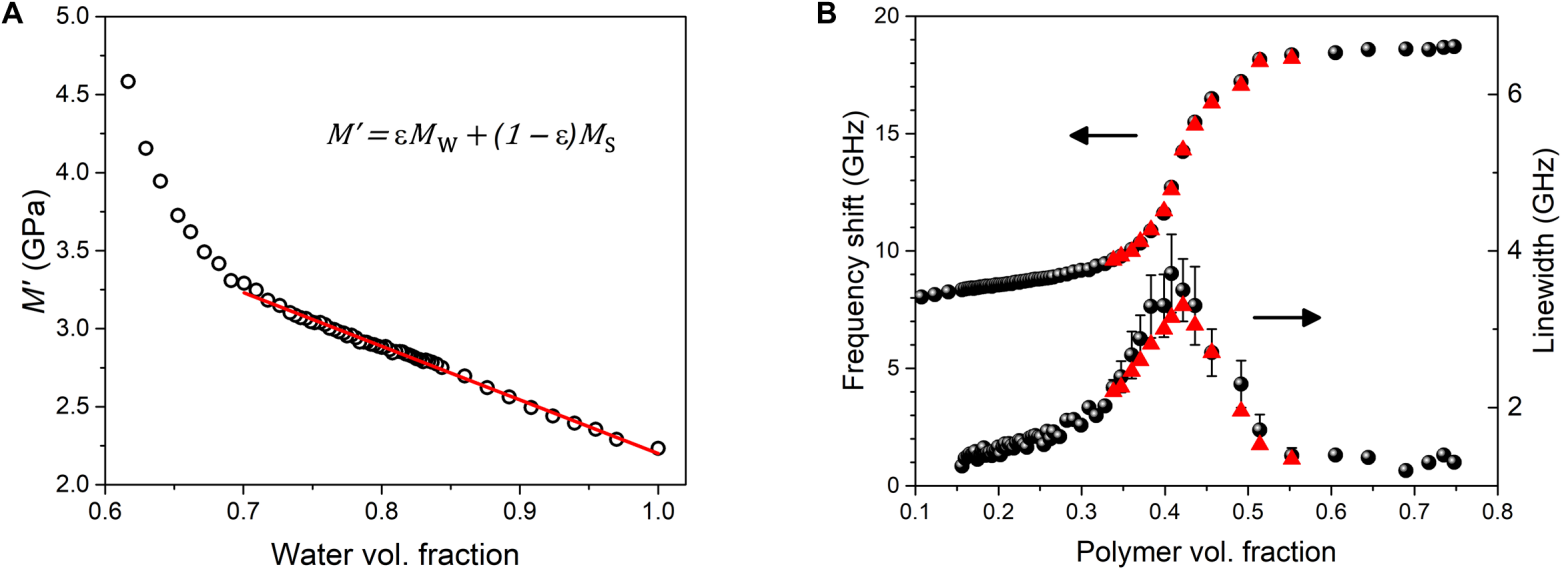
Low hydration Brillouin spectroscopy. Evolution in (**A**) storage modulus versus water volume fraction ε. Linear fit to a Voigt model (equation in figure) yields the elastic moduli of water and solute: *M*_w_= 2.20 GPa and *M*_s_ = 5.64 GPa; *R*^2^ = 0.994. (**B**) (Top plot) Brillouin frequency shift and (bottom plot) linewidth of gelatin versus polymer volume fraction. The error bars encompass the range of values obtained from the fits. The red triangles denote the frequency shifts and linewidths of the theoretical curves derived from the viscoelastic fit.

The evolution in storage modulus at constant temperature (Fig. 1D) shows only a 20% increase compared with 89% for the loss modulus. This is to be expected, because the modulus of water is high (2.2 GPa) and the addition of polymer produces only a small effect that, based on the rule of mixing, can be modeled as a weighted average of solvent and solute moduli (Fig. 3A). A fit to the Voigt model (*45*) across a broad range of concentrations (from 100 to 70% water content) yields a solute modulus of 5.64 GPa, which is plausible for a highly hydrated network of collagen molecules. The Voigt model was found to fit the data better than the inverse relation (Reuss) used in previous works.

This result shows that in this system, the Brillouin frequency shift is sensitive to the presence of the polymer network and, furthermore, that the modulus of the network can be determined provided that the spectrometer has adequate resolution.

The Brillouin frequency shift is so sensitive to the elastic properties of the network that it reveals the onset of a sol-gel transition, which arises from the development of a percolative cluster involving a population of cross-linked collagen molecules. We observed this phenomenon by investigating two different gel concentrations, 10 and 20%, as a function of temperature. The sol-gel transition gives rise to a small “step” gradient of the frequency shift (see arrows in fig. S4A). In contrast, the change in linewidth shows no discontinuity at the gel transition point (fig. S4B). This is consistent with the fact that, even in the gel phase, a major fraction of molecules are still in the liquid phase and that their diffusive motion dominates the picosecond dynamics, giving rise to the broadening of the Brillouin spectra in both the liquid and gel phases. These motions are arrested at the glass transition. The effect of a sol-gel transition on the loss and storage moduli is schematically depicted in Fig. 2B for a polymer concentration high enough (or temperature low enough) to produce a transition to the gel state. The solid-like portion of the sample, composed of cross-linked collagen molecules, is responsible for the relaxation process occurring at very long time scales (hundreds of seconds or more), and this gives rise to the divergence of the “static” viscosity and the onset of shear (*G*) and Young’s (*E*) moduli. The small number of molecules involved in this process is responsible for two phenomena: (i) a state in which the values of *G* and *E* are orders of magnitude smaller than the longitudinal modulus [note that the Young’s moduli derived from compressive testing of these hydrogels are of the order of kilopascal (fig. S5), while the high-frequency longitudinal moduli are in the gigapascal range], and (ii) a small jump in the value of *M*′ (from dashed to solid line in Fig. 2B), revealed by the experiments reported in fig. S4A, and a smooth transition in *M*′′, as shown in fig. S4B, because the liquid fraction of the sample is almost unchanged by the gelation process and is still responsible for the high-frequency (picosecond) relaxation, which is comparable to that of the diluted solutions (Fig. 2A).

### Low hydration

As the water fraction in the samples is reduced, the dynamics of the residual water are increasingly coupled to those of the collagen molecules until an arrested (glassy) phase is attained. Note that this is the mechanism of hardening of animal glue, one of the most widely used glues worldwide. In fact, the word collagen derives from the Greek “kolla,” glue. The transition from the liquid phase (low elastic modulus) to the solid phase (high elastic modulus) is revealed by the frequency dispersion and the associated maximum in linewidth of the Brillouin data (Fig. 3B).

**Fig. 4:**
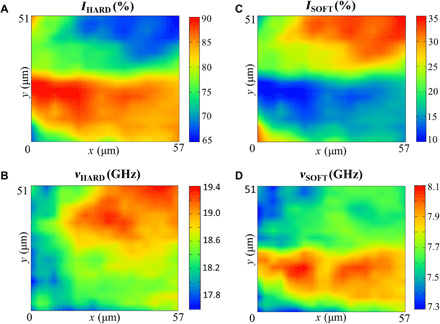
Brillouin microscopy of articular cartilage. Maps based on (**A** and **C**) intensity and (**B** and **D**) frequency shift derived from spectral moment analysis (see Materials and Methods). Hard and soft components denote distinct parts of cartilage corresponding to high- and low-frequency Brillouin peaks, 19 and 8 GHz, and are attributed to collagen fiber bundles and matrix-dispersed collagen, respectively.

Regarding the frequency shift, the two limiting cases of liquid- and solid-like gels reproduce remarkably well the Brillouin response of articular cartilage (Fig. 4) (*46*) with a peak at 8 GHz, which is characteristic of the soft component (disordered phase of thin, poorly oriented collagen fibers, proteoglycans, and water), and a peak at 19 GHz related to the hard component (collagen fiber bundles).Hence, our model system mimics the soft component of cartilage in the hydrated condition and the hard component of cartilage, namely, the collagen fiber bundles, in the dehydrated condition. 

The glass transition in dried collagen was first investigated through a more traditional thermodynamic path by Flory and Garret (*47*) and attributed to the temperature-induced arrest of the amorphous fraction and of the side chains of collagen molecules. As in analyses of the hardening of epoxy resins (*48*, *49*), the mode-coupling theory (MCT) (*50*) provides a rationale for the early stage of the structural arrest associated with the glass transition of collagen (see Materials and Methods). We stress that the time scale investigated by BLS is the most appropriate to unveil the divergence of the structural relaxation that is the intimate nature of the glass transition itself. In fact, fundamental physical studies on the glass transition phenomenon have shown that the relaxation of density fluctuations in the gigahertz region is dominated by the coupling between density fluctuation modes, originating the glass transition itself (*50*).

**Fig. 5:**
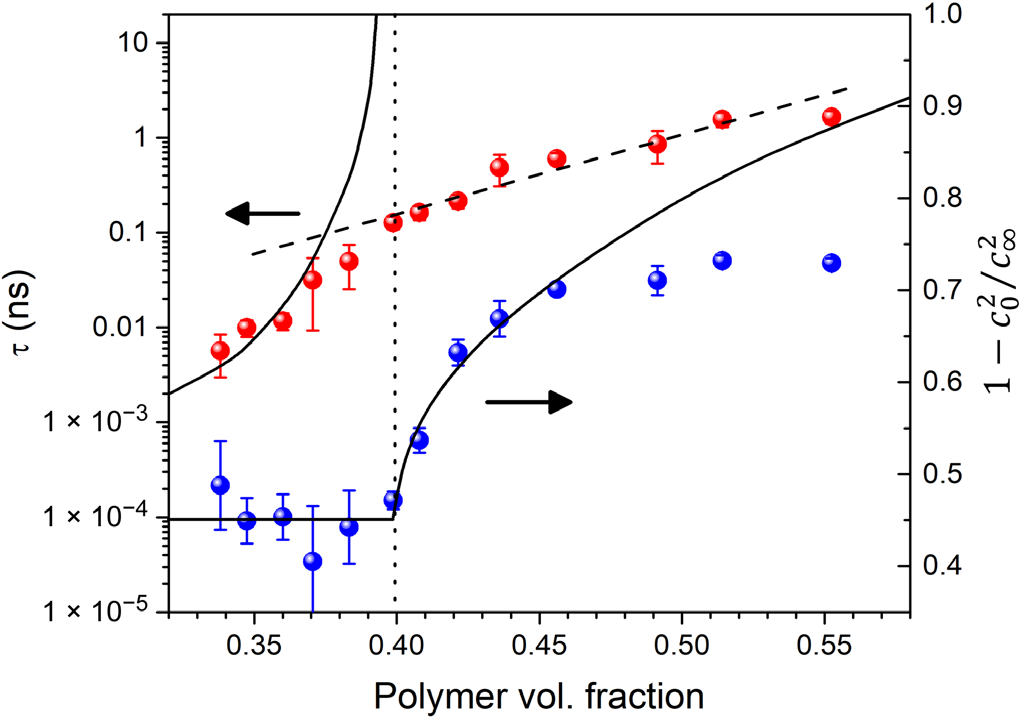
Concentration dependence of the relaxation time and nonergodicity parameter. Plot of the relaxation time τ and nonergodicity parameter $f=1-c_{0}^{2}/c_{\infty }^{2}$ versus polymer volume fraction *x*. Deviations of data points (full circles) from ideal behavior (solid lines) can be explained by secondary relaxation processes, which are relevant for real systems. Dashed line is a guide for the eye, and dotted line denotes the ideal critical concentration for the structural arrest predicted by the MCT.

Figure 2C qualitatively shows the effect of progressive dehydration as the structural relaxation shifts to lower frequencies. As deduced by Eq. 3 (see Materials and Methods), the condition of maximum linewidth is reached when the maximum of *M*′′(ω) matches the frequency of the Brillouin peak (dotted curve in Fig. 2C). Further reduction in hydration (full line in Fig. 2C) gives rise to a reduction in linewidth and an increase in Brillouin frequency shift approaching the solid-like (unrelaxed) condition ${M_{\infty }}^{\prime }=\rho c_{\infty }^{2}$. The full viscoelastic treatment of Brillouin spectra in this regime, the early stage of liquid-glass transition, by means of the MCT is reported in Materials and Methods, giving the red triangles in Fig. 3B. The signatures of singularity in the nonergodicity parameter ($1-c_{0}^{2}/c_{\infty }^{2}$) and of divergence of the structural relaxation (see Fig. 5), consistent with the predictions of MCT, suggest that the ergodicity breakdown described by MCT is a more “universal” mechanism of glass transition than ever supposed to be before.

In summary, Brillouin scattering has revealed

1) high sensitivity to small changes in storage modulus in diluted samples (gelation);

2) large changes in loss modulus in diluted samples due to hydrophobic hydration;

3) small increase in microviscosity up to 30 to 40% polymer concentration relative to static viscosity, which already diverged due to gelation. This confirms the possibility of diffusion of small molecules in macroscopically solid-like samples and provides a method for measuring viscosity on a microscale;

4) early stage of microscopic transition from liquid to solid state at 40% polymer fraction, well described by the MCT of glass transition;

5) insights into the physical states of cornea and cartilage that can lead to a better understanding of human diseases and potentially to improvements in health care.

## MATERIALS AND METHODS

### Hydrogel preparation

Hydrogels were prepared as previously described (*51*), from ~225 Bloom type-B bovine skin gelatin powder (G9382, Sigma-Aldrich) and distilled water in the concentration range 4 to 18% (w/w) gelatin. Gelatin powder and water were mixed under magnetic stirring while being heated in a water bath at 55° to 65°C for 60 min to ensure complete dissolution. Gels were left to cool to 40°C and were then transferred to storage vessels (see below), sealed to reduce evaporation, and then left to cool down to room temperature. Measurements were conducted approximately 24 hours after preparation, as preliminary testing established that this time was sufficient for the gel to stabilize.

### Refractive index and density measurements

The refractive index of all samples prepared was measured using an Abbe refractometer (Atago model NAR-1T liquid; resolution, 0.0002 nD), with distilled water as a reference. A small sample of gelatin was removed from the bulk before gelation (at ~40°C) and left to set in between the plates of the refractometer, ensuring good contact with both plates. Measurements were taken when the gel reached room temperature (fig. S2A).

Density was determined from a biphasic ideal mixing model

$$\rho =\frac{\left(m_{w}+m_{g}\right)}{\left(\frac{m_{w}}{\rho_{w}}+\frac{m_{g}}{\rho_{g}}\right)}$$ (1)

where *m*_w_ and *m*_g_ denote the mass of water and dry gelatin, respectively, and ρ_w_ and ρ_g_ are the corresponding mass densities, which were 1.00 g/cm^−3^ for water and 1.35 g/cm^3^ for dry gelatin (*52*). It is observed that the change in density-to-square refractive index ratio is less than 1% across the entire concentration range probed here (fig. S2B).

### Compressive testing

After preparation, the gels were transferred into custom-built aluminum molds. An Instron ElectroPuls E10000 (High Wycombe, UK) linear dynamic test instrument was used to perform unconfined compressive testing on the hydrogels at a steady rate of 0.03 mm/s. The cylindrical gel sample (21-mm diameter, ~10-mm thickness) sat on a flat aluminum base, and the applied load was measured by a 1-kN load cell (<2% linearity error) mounted onto a flat aluminum plate in contact with the top surface of the gel. Applied strain of up to 10% did not cause a substantial change in shape of the gels radially. Hence, from the uniaxial deformation, the gradient of the stress-strain curve was used to calculate the Young’s modulus of the hydrogel samples (fig. S5).

### Brillouin spectroscopy

Brillouin spectra of the hydrogels were acquired using a high-contrast tandem Fabry-Pérot (TFP-2 HC) interferometer system, with 532-nm continuous wave laser and a 20× [numerical aperture (NA), 0.42] objective, as previously described (*53*). Spectral resolution was 135 MHz, and contrast was >150 dB. Laser power at the sample was a few milliwatts, and acquisition time was 17 s per spectrum.

#### High hydration

Hydrogels prepared up to the solubility limit (18%) were transferred to glass vials, and measurements were conducted in triplicate using a backscattering geometry with 180° scattering angle. At two concentrations, 10 and 20% gelatin, measurements were also conducted as a function of temperature, from 65° to 4°–5°C (water bath), to encompass the sol-gel transition. Brillouin peaks were analyzed using a DHO function (*5*, *54*) in the range 7 to 9 GHz. Average fit parameters from Stokes and anti-Stokes peaks were obtained after deconvolution of the instrumental response function.

#### Low hydration

For hydrogel dehydration, a thin film (200- to 300-μm thickness) was deposited onto a reflective silicon substrate and positioned on an analytical scale (Sartorius BL210S) to monitor the change in concentration during the Brillouin measurements. Spectra were acquired according to a previously established protocol (*21*, *25*), using an achromatic lens (NA 0.033), and the sample was positioned at a 45° angle to the incident beam with a laser power of ~35 mW. Both bulk and parallel-to-surface acoustic modes were detected in this geometry (see Supplementary Methods and fig. S6).

#### Longitudinal elastic moduli

The storage modulus *M*^′^ was derived from the Brillouin frequency shift ω_B_ through the relation

$$M\prime ={\left(\frac{\lambda }{4\pi }\right)}^{2}\frac{\rho }{n^{2}}{\omega_{B}}^{2}$$ (2)

where $\frac{{\lambda \omega }_{B}}{4\pi n}=c$ is the acoustic wave velocity, λ is the excitation wavelength (532 nm), and ρ and *n* are the mass density and refractive index of the medium, respectively. Equation 2 shows that there is a direct relation between the real part of the longitudinal modulus and the square Brillouin shift, through the ratio ρ/*n*^2^.

In a similar way, the loss modulus *M*^′′^ can be derived from the Brillouin frequency shift ω_B_ and linewidth Γ_B_

$$M\prime \prime ={\left(\frac{\lambda }{4\pi }\right)}^{2}\frac{\rho }{n^{2}}\omega_{B}\Gamma_{B}$$ (3)

Note that Eqs. 2 and 3 are valid for backscattering geometry, which is typical of microscopy applications, and the Brillouin shift is expressed in units of angular frequency.

#### Linearized model at high hydration

In the case of water and diluted aqueous solutions, the frequency of Brillouin lines is much smaller than the molecular relaxation rates (Fig. 2A). In this relaxed condition, the linewidth of Brillouin peaks Γ_B_ yields the “longitudinal” kinematic viscosity *b* of the liquid through the relationship *b* = ρΓ_B_/*q*^2^, where *q* is the exchanged wave vector, as already defined.

The longitudinal kinematic viscosity, in turn, can give information on the characteristic times of molecular relaxations. For aqueous solutions, density fluctuations are generally characterized by a two-step relaxation associated with hydration and bulk water with characteristic times, τ_h_ and τ_b_, respectively. The longitudinal viscosity can then be written as (*44*)

$$b={∆}_{c}[\alpha \tau_{h}+(1-\alpha )\tau_{b}]+b_{\infty }$$ (4)

where α is the fraction of relaxation strength associated to hydration water, *b*_∞_ accounts for contributions to viscosity that are very fast (instantaneous) relative to the picosecond time scale investigated by Brillouin scattering, and ∆*_c_* is the total relaxation strength of the solution at each polymer concentration, given by relaxed (*c*_0_) and unrelaxed (*c*_∞_) sound velocities through the relationship: ${∆}_{c}=c_{\infty }^{2}-c_{0}^{2}$. Assuming that the relaxation strengths of the two processes are proportional to the relative fractions of hydration and bulk water, we can express α = *f_r_N*_h_, where *f_r_* is the fraction of polymer-to-water molecules and *N*_h_ is the hydration number. Moreover, hydration water molecules are typically found to diffuse more slowly than bulk molecules, and the retardation parameter ε = τ_h_/τ_b_ was used to quantify this effect. Equation 4 can thus be rearranged as

$$\frac{{∆}_{0}}{{∆}_{c}}\frac{b-b_{\infty }}{b_{0}-b_{\infty }}-1=N_{h}(\varepsilon -1)f_{r}$$ (5)

where *b*_0_ and ∆_0_ are the kinematic viscosity and relaxation strength of pure water, with *c*_0_ obtained from the frequency position of Brillouin peaks and *c*_∞_= 2860 m/s measured by inelastic x-ray scattering (IXS) (*55*) and assumed to be independent of polymer concentration (*56*); *b*_∞_= 2.99 10^−3^ cm^2^/s was derived from IXS measurements (*55*).

The prediction of Eq. 5 for the change of viscosity of the solution as a function of polymer concentration is tested in fig. S3. A well-defined linear behavior of $\frac{{∆}_{0}}{{∆}_{c}}\frac{b-b_{\infty }}{b_{0}-b_{\infty }}-1$ versus *f_r_* can be seen across the whole range of polymer molecular fraction, and the linear fit of the data gives *N*_h_(ε − 1) = 9566 (± 290). We then used a combination of this result derived using a linearized model from hydrodynamic theory (*44*) and published data from MD simulations (*26*) to derive the retardation factor ε. The hydration number *N*_h_ can be estimated by geometrical arguments as the number of water molecules (number density ρ = 33.37·10^27^ m^−3^) within 3.1 Å of the triple helix surface, by modeling the collagen triple helix as a rod with a radius of 0.36 nm and a length of 300 nm (*26*), giving *N*_h_ = 10 k. From this value, a retardation factor ε = 1.9 can be deduced for hydration water, which is in the range previously found for hydrophobic hydration in a large class of biomimetic molecules (*44*, *56*). The close agreement between the prediction of our two-step relaxation model and the measured concentration dependence of Brillouin parameters, together with the adequate value obtained for the retardation of hydration water, supports the view that water forming the first hydration shell of collagen molecules has a great impact on the dynamics of these gelatins, thus affecting the viscosity and loss modulus far more than the elasticity and storage modulus.

#### Glass transition

The glass transition is a dynamic process that occurs as an abrupt increase in the structural relaxation time, leading the system out of equilibrium (ergodic to nonergodic transition) (*50*). In the framework of MCT, the transition is induced by a slowdown of density fluctuations, which, in the frequency domain, can be described by the complex frequency-dependent longitudinal modulus *M*(ω) = *M*′(ω) + *iM*′′(ω). Close to the glass transition, a stretched exponential relaxation of the longitudinal modulus typically occurs, described by the Kohlrausch-Williams-Watts (KWW) law: *e*^( − *t*/τ)^β^^, where τ is the characteristic time and β < 1 is the stretching parameter. In the frequency domain, the Fourier transform of the KWW law can be conveniently described by a Havriliak-Negami relaxation function (*57*)

$$\frac{M(\omega )}{\rho }=c_{\infty }^{2}-\frac{c_{\infty }^{2}-c_{0}^{2}}{{\left[1+{\left(i\omega \tau \right)}^{a}\right]}^{b}}$$ (6)

where *c*_0_ and *c*_∞_ are the relaxed (low frequency ω or low τ with respect to the Brillouin frequency, such as in the high hydration regime) and unrelaxed (high ω or high τ, low hydration) sound velocities; *a* and *b* are the stretching parameters determined by the value of the KWW β parameter (*57*). The MCT, in its basic formulation, predicts a power law divergence of the relaxation time τ ∝ ( − ε)^−γ^ and a square root singularity of the amplitude of the structural relaxation (nonergodicity parameter) $1-c_{0}^{2}/c_{\infty }^{2}=f_{q}=f_{q}^{c}+h_{q}\sqrt{\varepsilon }$ for the control parameter ε → 0. The nonergodicity parameter *f_q_* quantifies the arrest of density fluctuations in the nonergodic state. Depending on the experimental path, the control parameter can be expressed in terms of temperature (thermal vitrification) or density (pressure vitrification) or volume fraction of polymer molecules in the case of colloidal suspensions (*50*). Here, we define it in terms of volume fraction of collagen molecules *x* as ε = (*x* − *x*_0_)/*x*_0_, where *x*_0_ is the “ideal” critical concentration for the structural arrest. According to MCT, the values of the parameters regulating the stretching of the relaxation function and the power law divergence of the relaxation time depend only on the structure of the sample and are mutually related by well-defined analytical expressions (*58*). Measuring these parameters give a quantitative test of the predictions of the theory.

Brillouin spectra from longitudinal acoustic modes are informative of *M*(ω) and can be used to test the predictions of the MCT, since they give direct access to the spectrum of density fluctuations (fluctuation-dissipation theorem) (*59*)

$$I_{q}(\omega )=\frac{I_{0}}{\omega }I\{{\left[M(\omega )/\rho -\omega^{2}/q^{2}\right]}^{-1}\}$$ (7)

This equation, where I denotes the imaginary part, shows that the maximum of information (maximum intensity) in the Brillouin spectrum is at the resonance (Brillouin peak) occurring around ω_B_ = (*q*^2^*M*^′^(ω_B_)/ρ)^1/2^. Unfortunately, the fit of a single Brillouin spectrum to this equation is not sufficient to get the whole set of relaxation parameters *c*_0_, *c*_∞_, τ, and β. Different strategies can be implemented to mitigate this problem (*54*). In the present work, we expanded the frequency range by collecting light from two simultaneous scattering geometries (see the Supplementary Methods) and independently estimated the values of *n*, *c*_0_, and β, so that *c*_∞_ and τ were the only free parameters in fitting Brillouin spectra.

From the fit, the concentration dependence of the relaxation time τ and of the nonergodicity parameter $1-c_{0}^{2}/c_{\infty }^{2}$ was obtained and is reported in Fig. 5 to be compared with the predictions of the MCT.

Although the system investigated here is by far more complex than the liquids and colloidal suspensions usually analyzed with MCT, signatures of a critical concentration *x*_0_ located around 40% polymer can be clearly seen in Fig. 5. In particular, *f_q_* shows an inflection close to *x*_0_, with an increase at higher concentrations that mimics the square root behavior predicted by the theory (solid line). Moreover, at lower concentrations, τ follows the power law behavior predicted by the theory, with an exponent γ of approximately 4, the value related by MCT to the stretching parameter of the structural relaxation β = 0.45 (see the Supplementary Methods). The deviation from the power law visible in Fig. 5 when approaching the critical point is quite typical for all glass-forming systems (*50*) and attributed to the presence of additional (secondary) relaxation processes responsible for restoring ergodicity above *x_c_*.

As a whole, these results are in good agreement with the predictions of the MCT for the glass transition, which was previously verified in simple glass formers through more traditional thermodynamic paths, namely, with temperature as the control parameter (*50*). This was previously verified as a function of temperature (*60*, *61*) and pressure (*62*), upon hardening of epoxy glues (*49*) and astonishingly here in the hardening of “kolla” controlled by changing concentration.

#### Brillouin microscopy of articular cartilage

Brillouin maps of a cross section of articular cartilage were acquired using the same setup described above (see “Brillouin spectroscopy” section) by a 2-μm step raster scan of the human femoral head sample already reported in (*46*). Brillouin peaks were analyzed using the spectral moment method (*63*) in the range 4 to 13 GHz and 13 to 32 GHz for the low-frequency and high-frequency modes, respectively. Intensity and frequency shift of the peaks derived from this analysis are plotted and displayed in Fig. 4.

## Supplementary Material

abc1937_SM.pdf

## SUPPLEMENTARY MATERIALS

Supplementary material for this article is available at http://advances.sciencemag.org/cgi/content/full/6/44/eabc1937/DC1

## Appendix

View/request a protocol for this paper from *Bio-protocol*.
