# Supplementary material for: Viscoelastic properties of biopolymer hydrogels determined by Brillouin spectroscopy: A probe of tissue micromechanics
Source: Sci Adv. 2020 Oct 30;6(44):eabc1937. doi: 10.1126/sciadv.abc1937 (PMC7608813; doi:10.1126/sciadv.abc1937)
Supplement: abc1937_SM.pdf [file abc1937_SM.pdf]

Science Advances
AAAS

advances.sciencemag.org/cgi/content/full/6/44/eabc1937/DC1

Supplementary Materials for

Viscoelastic properties of biopolymer hydrogels determined by Brillouin spectroscopy: A probe of tissue micromechanics

Michelle Bailey, Martina Alunni-Cardinali, Noemi Correa, Silvia Caponi, Timothy Holsgrove, Hugh Barr, Nick Stone, C. Peter Winlove, Daniele Fioretto*, Francesca Palombo*

*Corresponding author. Email: f.palombo@exeter.ac.uk (F.P.); daniele.fioretto@unipg.it (D.F.)

Published 30 October 2020, *Sci. Adv.* **6**, eabc1937 (2020)
DOI: 10.1126/sciadv.abc1937

This PDF file includes:

Figs. S1 to S9
Supplementary Methods
References

Supplementary Materials

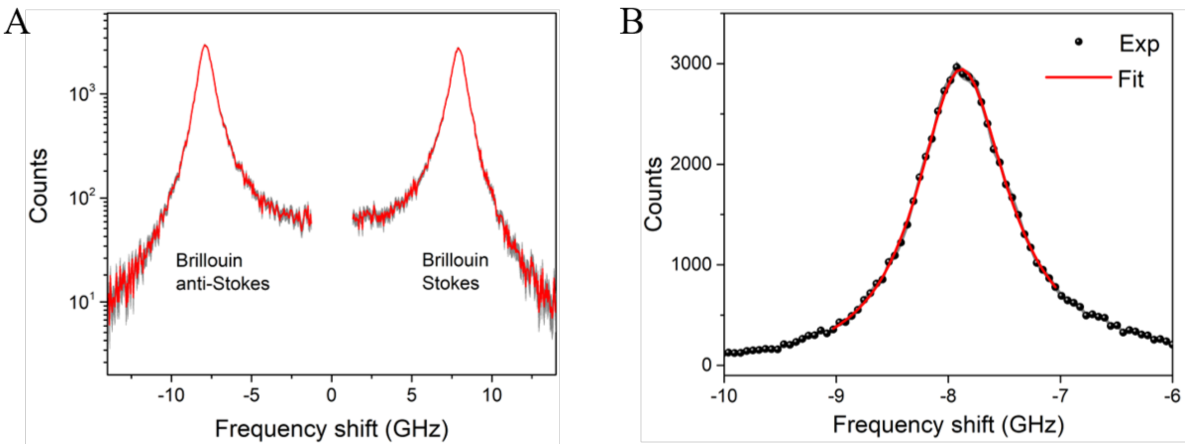

Supplementary Figure s1. Brillouin spectrum and fit. (A) Brillouin spectrum of a 12% gelatin hydrogel, with grey shading denoting the standard error (square root of number of counts). (B) Results of a damped harmonic oscillator (DHO) fit (see eq. S2) to the Stokes peak.

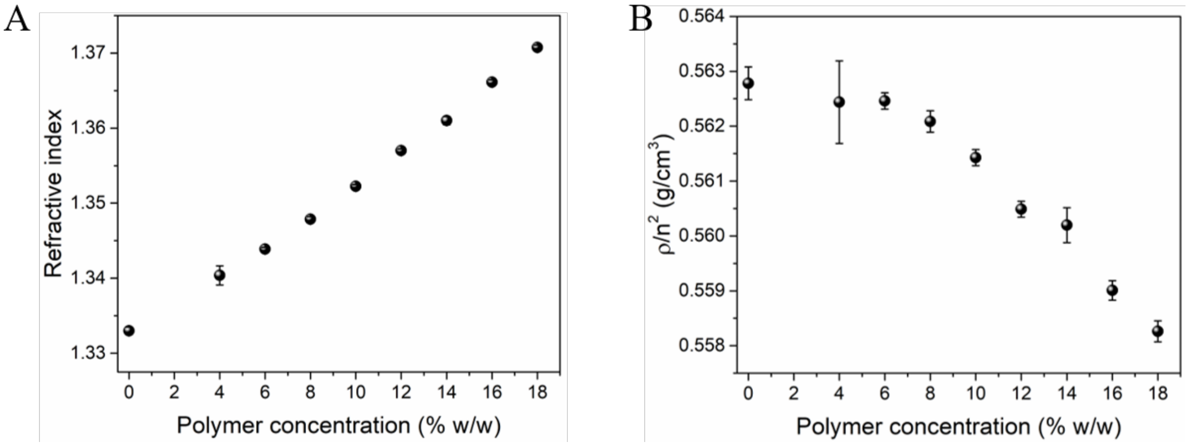

Supplementary Figure s2. Refractive index and density. (A) Measured refractive index for varying gelatin concentration. Error bars indicate the standard deviation for four measurements on different gels. (B) Plot of the density-to-refractive index square ratio vs. polymer concentration. The maximum deviation observed here is <1%, validating the approximation of uniform ratio made in many Brillouin scattering investigations.

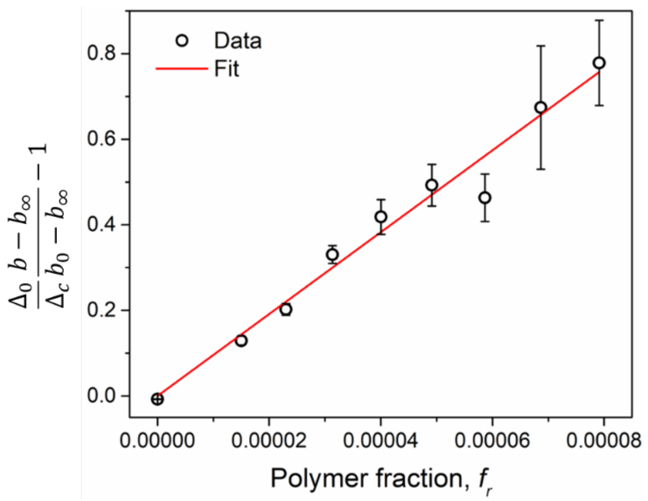

Supplementary Figure s3. Linearized model. Plot of Eq. 4 (empty circles) and linear fit (red line) giving a gradient $N_h(\varepsilon - 1)$ = 9566 (see main text; Methods).

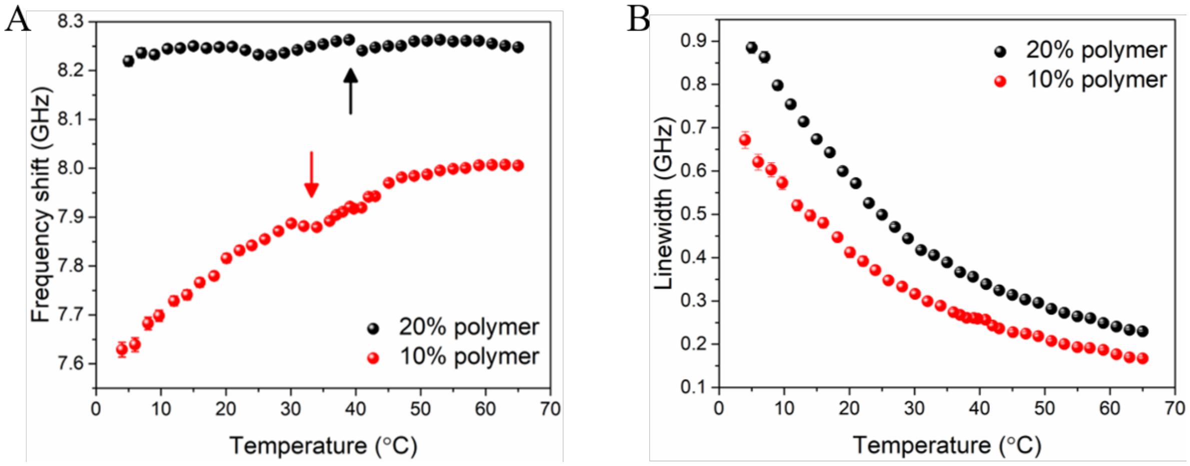

Supplementary Figure s4. Gel transition. Evolution of (A) frequency shift and (B) linewidth derived from Brillouin spectra of hydrogels as the temperature is reduced from 65 to 4-5°C. Arrows indicate the gel transition.

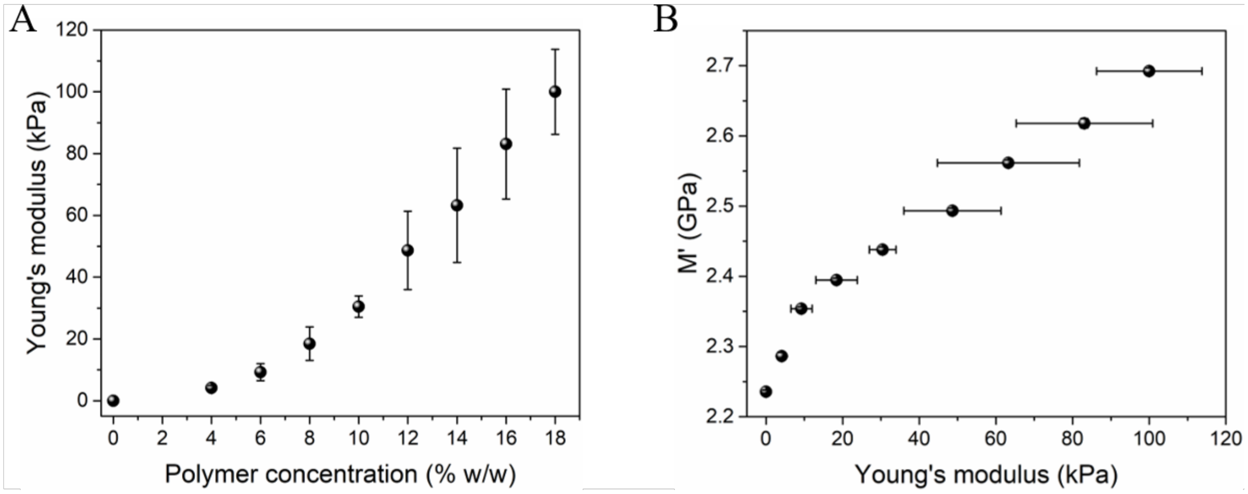

Supplementary Figure s5. Compressive testing measurements. (A) Plot of Young's modulus vs. polymer concentration. (B) Plot of longitudinal modulus vs. Young's modulus for all the gelatins. Error bars denote the standard deviation.

Supplementary Methods

To increase the information in each spectrum, we enlarged the investigated frequency range by mounting the sample onto a reflecting substrate (fig. S6), so that the scattering from phonons travelling parallel to the surface was simultaneously collected, corresponding to a scattering wavevector $q_S = 2k_0 \sin\theta/2$ (*21*).

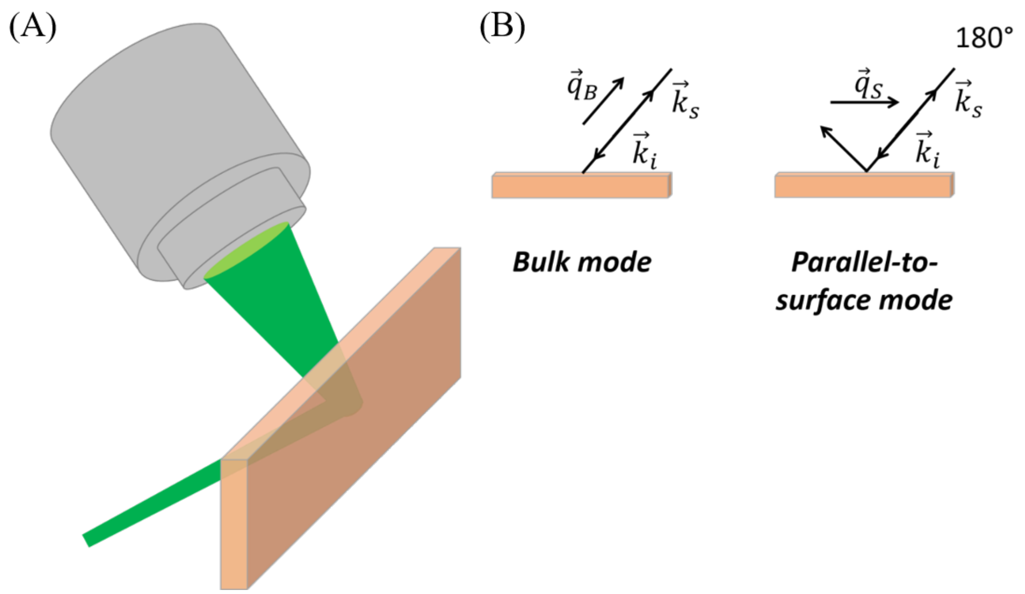

Supplementary Figure s6. 45° scattering geometry. (A) Schematic diagram of the platelet-like configuration employed to access both bulk and parallel to surface modes in Brillouin spectroscopy measurements at a 45° angle of the incident beam to the sample mounted onto a reflective substrate. (B) Diagrams of wavevectors in the scattering process.

In this case, the measured spectrum is given by the sum of two Brillouin peaks:

$$I_q^{TOT}(\omega) = I_{q_S}(\omega) + I_{q_B}(\omega) \quad \text{(s1)}$$

where subscripts q_S and q_B refer to the parallel to surface and bulk modes, respectively. Further improvement to the fitting procedure was obtained by fixing $n(x)$, $c_0(x)$ and $\beta(x)$ to values obtained from extrapolation of limiting behaviours, as described below.

In a narrow region around the frequency of Brillouin peaks ω_B, one can approximate the spectrum of density fluctuations (see main text) to a DHO function (*54*):

$$I_q(\omega) = I_0 \frac{\omega_B^2 \Gamma_B}{\left(\omega_B^2 - \omega^2\right)^2 - (\omega \Gamma_B)^2} \quad \text{(s2)}$$

where $\omega_B^2 = q^2 M'(\omega_B)/\rho$ and $\omega\Gamma_B = q^2 M''(\omega_B)/\rho + \omega\Gamma_\infty$, with $M'(\omega_B)$ and $M''(\omega_B)$ being the real and imaginary parts of the modulus at the single frequency of the Brillouin peak, and $\Gamma_\infty$the unrelaxed part of kinematic viscosity.

In both relaxed and unrelaxed conditions, the modulus is independent of frequency, and both bulk and parallel to surface modes give the same value for M'. This implies that, in these conditions, $\omega_{0B}/q_B = \omega_{0S}/q_S$ from which the refractive index can be obtained, $n = (\omega_{0B}/\omega_{0S}) \sin\theta/2$. The values of n measured by refractometry at low concentrations (black dots) are reported in fig. S7 together with the values obtained by the ratio of the frequencies of bulk and parallel to surface modes (red dots).

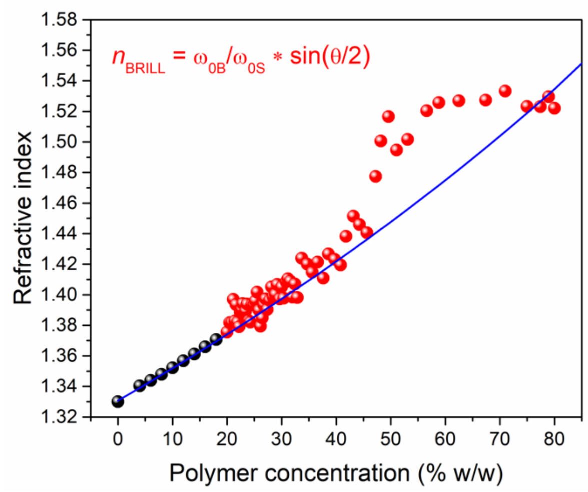

Supplementary Figure s7. Refractive index. Plot of the refractive index measured by refractometry at low concentration (black dots) and obtained from Brillouin measurements (red dots). The blue line is a linear extrapolation of $1/n^2$ in the range 0–19% polymer concentration, $n = 1/\sqrt{0.56467 - 0.00175x}$.

As expected, Brillouin data confirm the refractometry data in relaxed (low x) and unrelaxed (high x) conditions, validating the linear extrapolation of $1/n^2$. The rationale for this linear extrapolation can further be found in an almost constant ratio n^2/ρ (fig. S2B).

In the following elaboration, we will fix $n(x)$ according to this law.

Notice that in the intermediate x region, the presence of the relaxation process is associated with the frequency dependence of the modulus, responsible for the breakdown of the simple relation $n = (\omega_{0B}/\omega_{0S}) \sin\theta/2$ and the deviation from the linear behaviour observed in Fig. 3A. In this condition, Brillouin peaks will be most sensitive to the values of the relaxation parameters.

The next step for the characterization of the glass transition is the determination of the relaxed sound velocity, $c_0(x)$.

Using the Voigt fit described in the main text (Fig. 3A), we obtained values for the longitudinal modulus in this relaxed regime, $M_0(x)$. The concentration dependence of c_0 is then obtained as $c_0(x) = (M_0(x)/\rho)^{1/2}$.

A reasonable estimation for the value of the stretching parameter β can be obtained from the Cole-Cole plot of the imaginary vs. real part of the elastic modulus (*64*), shown in fig. S8.

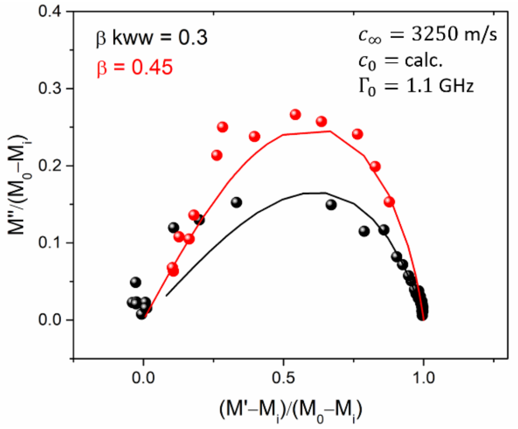

Supplementary Figure s8. Cole-Cole plot of the imaginary vs. real part of the elastic moduli. M' and M'' were obtained from DHO fit of the BLS peaks of bulk phonons. By an iterative process, in first approximation black dots were calculated fixing c_∞ to the limiting high concentration value c_∞ = 3250 m/s and subtracting a constant unrelaxed contribution Γ_∞=1.1 GHz from the measured linewidths. In this representation, the single exponential relaxation would give a semicircle. Conversely, the shrunk shape of the curve is evidence of a stretched exponential behaviour. A good representation of the data can be obtained using a stretching parameter $\beta \approx 0.3$ (solid line). This value was fixed to fit Brillouin spectra to eqs. S1, S2, and 6. A better approximation for $c_\infty(x)$ was thus obtained and used, in the second iteration, to recalculate the Cole-Cole plot (red dots), giving a β parameter of 0.45. This value was ultimately used to fit the Brillouin spectra.

Fixing the values of ρ, n, c_0 as described above and β = 0.45 as explained in fig. S8, we can now fit Brillouin spectra, both bulk and parallel to surface modes, to eqs. S1, S2, and 6, leaving only c_∞ and τ as free parameters. The results for the gels at x = 41% and 59% are shown in fig. S9.

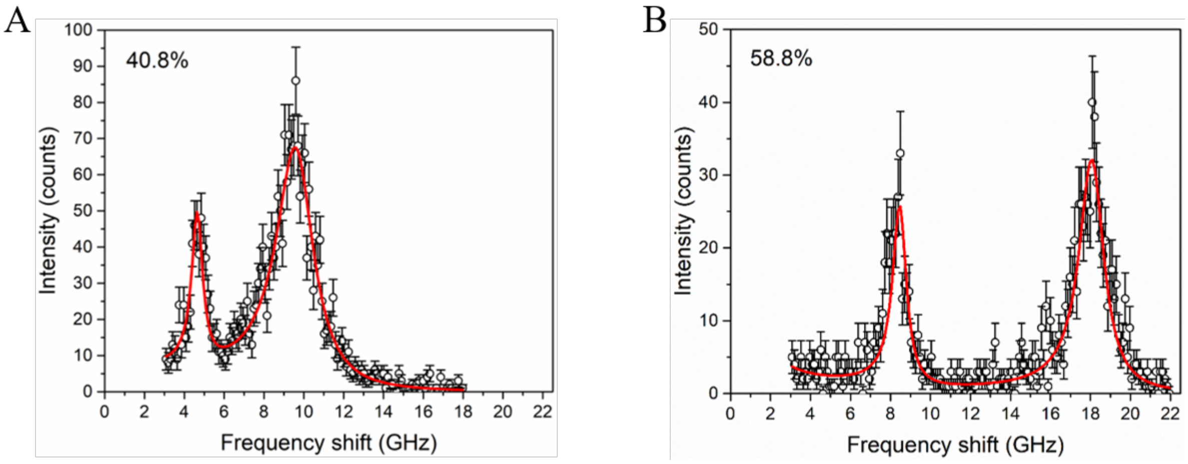

Supplementary Figure s9. Damped harmonic oscillator fit. Results of DHO fitting applied to both bulk (high frequency) and parallel-to-surface modes (low frequency) in Brillouin spectroscopy measurements at 45° of hydrogels at (A) 41% and (B) 59% polymer concentration.

REFERENCES AND NOTES

1. K. H. Vining, D. J. Mooney, Mechanical forces direct stem cell behaviour in development and regeneration. *Nat. Rev. Mol. Cell Biol.* **18**, 728–742 (2017).

2. V. Swaminathan, K. Mythreye, E. T. O'Brien, A. Berchuck, G. C. Blobe, R. Superfine, Mechanical stiffness grades metastatic potential in patient tumor cells and in cancer cell lines. *Cancer Res.* **71**, 5075–5080 (2011).

3. J. M. Northcott, I. S. Dean, J. K. Mouw, V. M. Weaver, Feeling stress: The mechanics of cancer progression and aggression. *Front. Cell Dev. Biol.* **6**, 17 (2018).

4. L. Brillouin, Diffusion de la lumière et des rayonnes X par un corps transparent homogène; influence de l'agitation thermique. *Ann. Phys.* **17**, 88–122 (1922).

5. F. Palombo, D. Fioretto, Brillouin light scattering: Applications in biomedical sciences. *Chem. Rev.* **119**, 7833–7847 (2019).

6. G. Scarcelli, W. J. Polacheck, H. T. Nia, K. Patel, A. J. Grodzinsky, R. D. Kamm, S. H. Yun, Noncontact three-dimensional mapping of intracellular hydromechanical properties by Brillouin microscopy. *Nat. Methods* **12**, 1132–1134 (2015).

7. S. Mattana, M. Mattarelli, L. Urbanelli, K. Sagini, C. Emiliani, M. D. Serra, D. Fioretto, S. Caponi, Non-contact mechanical and chemical analysis of single living cells by microspectroscopic techniques. *Light Sci. Appl.* **7**, 17139 (2018).

8. J. Margueritat, A. Virgone-Carlotta, S. Monnier, H. Delanoë-Ayari, H. C. Mertani, A. Berthelot, Q. Martinet, X. Dagany, C. Rivière, J.-P. Rieu, T. Dehoux, High-frequency mechanical properties of tumors measured by Brillouin light scattering. *Phys. Rev. Lett.* **122**, 018101 (2019).

9. K. Elsayad, S. Werner, M. Gallemí, J. Kong, E. R. S. Guajardo, L. Zhang, Y. Jaillais, T. Greb, Y. Belkhadir, Mapping the subcellular mechanical properties of live cells in tissues with fluorescence emission–Brillouin imaging. *Sci. Signal.* **9**, rs5 (2016).

10. G. Antonacci, V. de Turris, A. Rosa, G. Ruocco, Background-deflection Brillouin microscopy reveals altered biomechanics of intracellular stress granules by ALS protein FUS. *Commun. Biol.* **1**, 139 (2018).

11. R. Schlüßler, S. Möllmert, S. Abuhattum, G. Cojoc, P. Müller, K. Kim, C. Möckel, C. Zimmermann, J. Czarske, J. Guck, Mechanical mapping of spinal cord growth and repair in living zebrafish larvae by Brillouin imaging. *Biophys. J.* **115**, 911–923 (2018).

12. I. Remer, R. Shaashoua, N. Shemesh, A. Ben-Zvi, A. Bilenca, High-sensitivity and high-specificity biomechanical imaging by stimulated Brillouin scattering microscopy. *Nat. Methods* **17**, 913–916 (2020).

13. C. Bevilacqua, H. Sánchez-Iranzo, D. Richter, A. Diz-Muñoz, R. Prevedel, Imaging mechanical properties of sub-micron ECM in live zebrafish using Brillouin microscopy. *Biomed. Opt. Express* **10**, 1420–1431 (2019).

14. G. Antonacci, R. M. Pedrigi, A. Kondiboyina, V. V. Mehta, R. de Silva, C. Paterson, R. Krams, P. Török, Quantification of plaque stiffness by Brillouin microscopy in experimental thin cap fibroatheroma. *J. R. Soc. Interface* **12**, 20150843 (2015).

15. S. Mattana, S. Caponi, F. Tamagnini, D. Fioretto, F. Palombo, Viscoelasticity of amyloid plaques in transgenic mouse brain studied by Brillouin microspectroscopy and correlative Raman analysis. *J. Innov. Opt. Health Sci.* **10**, 1742001 (2017).

16. P. Shao, T. G. Seiler, A. M. Eltony, A. Ramier, S. J. J. Kwok, G. Scarcelli, R. Pineda II, S.-H. Yun, Effects of corneal hydration on Brillouin microscopy in vivo. *Invest. Ophthalmol. Vis. Sci.* **59**, 3020–3027 (2018).

17. R. Mercatelli, S. Mattana, L. Capozzoli, F. Ratto, F. Rossi, R. Pini, D. Fioretto, F. S. Pavone, S. Caponi, R. Cicchi, Morpho-mechanics of human collagen superstructures revealed by all-optical correlative micro-spectroscopies. *Commun. Biol.* **2**, 117 (2019).

18. E. Pukhlyakova, A. J. Aman, K. Elsayad, U. Technau, β-Catenin–dependent mechanotransduction dates back to the common ancestor of Cnidaria and Bilateria. *Proc. Natl. Acad. Sci. U.S.A.* **115**, 6231–6236 (2018).

19. R. M. Gouveia, G. Lepert, S. Gupta, R. R. Mohan, C. Paterson, C. J. Connon, Assessment of corneal substrate biomechanics and its effect on epithelial stem cell maintenance and differentiation. *Nat. Commun.* **10**, 1496 (2019).

20. K. J. Koski, P. Akhenblit, K. McKiernan, J. L. Yarger, Non-invasive determination of the complete elastic moduli of spider silks. *Nat. Mater.* **12**, 262–267 (2013).

21. F. Palombo, C. P. Winlove, R. S. Edginton, E. Green, N. Stone, S. Caponi, M. Madami, D. Fioretto, Biomechanics of fibrous proteins of the extracellular matrix studied by Brillouin scattering. *J. R. Soc. Interface* **11**, 20140739 (2014).

22. J. Randall, J. M. Vaughan, S. Cusak, Brillouin scattering in systems of biological significance [and discussion]. *Philos. Trans. R. Soc. A* **293**, 341–348 (1979).

23. S. Cusack, A. Miller, Determination of the elastic constants of collagen by Brillouin light scattering. *J. Mol. Biol.* **135**, 39–51 (1979).

24. S. Cusack, S. Lees, Variation of longitudinal acoustic velocity at gigahertz frequencies with water content in rat-tail tendon fibers. *Biopolymers* **23**, 337–351 (1984).

25. R. S. Edginton, S. Mattana, S. Caponi, D. Fioretto, E. Green, C. P. Winlove, F. Palombo, Preparation of Extracellular Matrix Protein Fibers for Brillouin Spectroscopy. *J. Vis. Exp.*, e54648 (2016).

26. S. Varma, J. P. R. O. Orgel, J. D. Schieber, Nanomechanics of type I collagen. *Biophys. J.* **111**, 50–56 (2016).

27. R. S. Edginton, E. M. Green, C. P. Winlove, D. Fioretto, F. Palombo, Dual scale biomechanics of extracellular matrix proteins probed by Brillouin scattering and quasistatic tensile testing. *Proc. SPIE BiOS*, 105040J (2018).

28. S. Caponi, C. Canale, O. Cavalleri, M. Vassalli, in *Nanotechnology Characterization Tools for Tissue Engineering and Medical Therapy*, C. S. S. R. Kumar, Ed. (Springer-Verlag GmbH Germany, 2019), chap. 2, pp. 69–111.

29. R. Prevedel, A. Diz-Muñoz, G. Ruocco, G. Antonacci, Brillouin microscopy: An emerging tool for mechanobiology. *Nat. Methods* **16**, 969–977 (2019).

30. V. C. Mow, C. C. Wang, C. T. Hung, The extracellular matrix, interstitial fluid and ions as a mechanical signal transducer in articular cartilage. *Osteoarthr. Cartil.* **7**, 41–58 (1999).

31. C. Frantz, K. M. Stewart, V. M. Weaver, The extracellular matrix at a glance. *J. Cell Sci.* **123**, 4195–4200 (2010).

32. M. Guo, A. F. Pegoraro, A. Mao, E. H. Zhou, P. R. Arany, Y. Han, D. T. Burnette, M. H. Jensen, K. E. Kasza, J. R. Moore, F. C. Mackintosh, J. J. Fredberg, D. J. Mooney, J. Lippincott-Schwartz, D. A. Weitz, Cell volume change through water efflux impacts cell stiffness and stem cell fate. *Proc. Natl. Acad. Sci. U.S.A.* **114**, E8618–E8627 (2017).

33. Y. L. Han, A. F. Pegoraro, H. Li, K. Li, Y. Yuan, G. Xu, Z. Gu, J. Sun, Y. Hao, S. K. Gupta, Y. Li, W. Tang, H. Kang, L. Teng, J. J. Fredberg, M. Guo, Cell swelling, softening and invasion in a three-dimensional breast cancer model. *Nat. Phys.* **16**, 101–108 (2020).

34. M. Urbanska, H. E. Muñoz, J. S. Bagnall, O. Otto, S. R. Manalis, D. D. Carlo, J. Guck, A comparison of microfluidic methods for high-throughput cell deformability measurements. *Nat. Methods* **17**, 587–593 (2020).

35. K. M. Stroka, H. Jiang, S.-H. Chen, Z. Tong, D. Wirtz, S. X. Sun, K. Konstantopoulos, Water permeation drives tumor cell migration in confined microenvironments. *Cell* **157**, 611–623 (2014).

36. P.-J. Wu, I. V. Kabakova, J. W. Ruberti, J. M. Sherwood, I. E. Dunlop, C. Paterson, P. Török, D. R. Overby, Water content, not stiffness, dominates Brillouin spectroscopy measurements in hydrated materials. *Nat. Methods* **15**, 561–562 (2018).

37. S. V. Adichtchev, Y. A. Karpegina, K. A. Okotrub, M. A. Surovtseva, V. A. Zykova, N. V. Surovtsev, Brillouin spectroscopy of biorelevant fluids in relation to viscosity and solute concentration. *Phys. Rev. E* **99**, 062410 (2019).

38. J. E. Chambers, M. Kubánková, R. G. Huber, I. López-Duarte, E. Avezov, P. J. Bond, S. J. Marciniak, M. K. Kuimova, An optical technique for mapping microviscosity dynamics in cellular organelles. *ACS Nano* **12**, 4398–4407 (2018).

39. E. H. Zhou, X. Trepat, C. Y. Park, G. Lenormand, M. N. Oliver, S. M. Mijailovich, C. Hardin, D. A. Weitz, J. P. Butler, J. J. Fredberg, Universal behavior of the osmotically compressed cell and its analogy to the colloidal glass transition. *Proc. Natl. Acad. Sci.* **106**, 10632–10637 (2009).

40. A. A. Hyman, C. A. Weber, F. Jülicher, Liquid-liquid phase separation in biology. *Ann. Rev. Cell Dev. Biol.* **30**, 39–58 (2014).

41. O. Lieleg, J. Kayser, G. Brambilla, L. Cipelletti, A. R. Bausch, Slow dynamics and internal stress relaxation in bundled cytoskeletal networks. *Nat. Mater.* **10**, 236–242 (2011).

42. A. Bot, R. P. C. Schram, G. H. Wegdam, Brillouin light scattering from a biopolymer gel: Hypersonic sound waves in gelatin. *Colloid Polym. Sci.* **273**, 252–256 (1995).

43. D. S. Bedborough, D. A. Jackson, Brillouin scattering study of gelatin gel using a double passed Fabry-Perot spectrometer. *Polymer* **17**, 573–576 (1976).

44. L. Comez, L. Lupi, M. Paolantoni, F. Picchiò, D. Fioretto, Hydration properties of small hydrophobic molecules by Brillouin light scattering. *J. Chem. Phys.* **137**, 114509 (2012).

45. T. W. Clyne, P. J. Withers, in *An Introduction to Metal Matrix Composites* (Cambridge Univ. Press, Cambridge, 1993), pp. 12–43.

46. M. A. Cardinali, D. Dallari, M. Govoni, C. Stagni, F. Marmi, M. Tschon, S. Brogini, D. Fioretto, A. Morresi, Brillouin micro-spectroscopy of subchondral, trabecular bone and articular cartilage of the human femoral head. *Biomed. Opt. Express* **10**, 2606–2611 (2019).

47. P. J. Flory, R. R. Garrett, Phase transitions in collagen and gelatin systems. *J. Am. Chem. Soc.* **80**, 4836–4845 (1958).

48. S. Corezzi, D. Fioretto, P. Rolla, Bond-controlled configurational entropy reduction in chemical vitrification. *Nature* **420**, 653–656 (2002).

49. S. Corezzi, L. Comez, G. Monaco, R. Verbeni, D. Fioretto, Bond-induced ergodicity breakdown in reactive mixtures. *Phys. Rev. Lett.* **96**, 255702 (2006).

50. W. Gotze, L. Sjogren, Relaxation processes in supercooled liquids. *Rep. Prog. Phys.* **55**, 241–376 (1992).

51. S. Kalyanam, R. D. Yapp, M. F. Insana, Poro-viscoelastic behavior of gelatin hydrogels under compression-implications for bioelasticity imaging. *J. Biomech. Eng.* **131**, 081005 (2009).

52. A. Taffel, CCXXXVI.—Thermal expansion of gelatin gels. *J. Chem. Soc. Trans.* **121**, 1971–1984 (1922).

53. F. Scarponi, S. Mattana, S. Corezzi, S. Caponi, L. Comez, P. Sassi, A. Morresi, M. Paolantoni, L. Urbanelli, C. Emiliani, L. Roscini, L. Corte, G. Cardinali, F. Palombo, J. R. Sandercock, D. Fioretto, High-performance versatile setup for simultaneous Brillouin-Raman microspectroscopy. *Phys. Rev. X* **7**, 031015 (2017).

54. L. Comez, C. Masciovecchio, G. Monaco, D. Fioretto, in *Solid State Physics*, E. C. Robert, L. S. Robert, Eds. (Academic Press, 2012), vol. 63, pp. 1–77.

55. G. Monaco, A. Cunsolo, G. Ruocco, F. Sette, Viscoelastic behavior of water in the terahertz-frequency range: An inelastic x-ray scattering study. *Phys. Rev. E* **60**, 5505–5521 (1999).

56. L. Lupi, L. Comez, C. Masciovecchio, A. Morresi, M. Paolantoni, P. Sassi, F. Scarponi, D. Fioretto, Hydrophobic hydration of tert-butyl alcohol studied by Brillouin light and inelastic ultraviolet scattering. *J. Chem. Phys.* **134**, 055104 (2011).

57. F. Alvarez, A. Alegra, J. Colmenero, Relationship between the time-domain Kohlrausch-Williams-Watts and frequency-domain Havriliak-Negami relaxation functions. *Phys. Rev. B* **44**, 7306–7312 (1991).

58. W. Gotze, The scaling functions for the β-relaxation process of supercooled liquids and glasses. *J. Phys. Condens. Matter* **2**, 8485–8498 (1990).

59. C. J. Montrose, V. A. Solovyev, T. A. Litovitz, Brillouin scattering and relaxation in liquids. *J. Acoust. Soc. Am.* **43**, 117–130 (1968).

60. S. Caponi, M. Zanatta, A. Fontana, L. E. Bove, L. Orsingher, F. Natali, C. Petrillo, F. Sacchetti, Ergodicity breaking in strong and network-forming glassy systems. *Phys. Rev. B* **79**, 172201 (2009).

61. F. Mallamace, C. Corsaro, N. Leone, V. Villari, N. Micali, S.-H. Chen, On the ergodicity of supercooled molecular glass-forming liquids at the dynamical arrest: The o-terphenyl case. *Sci. Rep.* **4**, 3747 (2014).

62. R. Casalini, M. Paluch, C. M. Roland, Dynamic crossover in supercooled liquids induced by high pressure. *J. Chem. Phys.* **118**, 5701–5703 (2003).

63. D. Fioretto, S. Caponi, F. Palombo, Brillouin-Raman mapping of natural fibers with spectral moment analysis. *Biomed. Opt. Express* **10**, 1469–1474 (2019).

64. N. G. McCrum, B. E. Read, G. Williams, *Anelastic and Dielectric Effects in Polymeric Solids* (Wiley, New York, 1967), 617.
